# Form, shape and function: segmented blood flow in the choriocapillaris

**DOI:** 10.1038/srep35754

**Published:** 2016-10-25

**Authors:** M. A. Zouache, I. Eames, C. A. Klettner, P. J. Luthert

**Affiliations:** 1 University College London, Institute of Ophthalmology, London, EC1V 9EL, United Kingdom; 2 University College London, Department of Mechanical Engineering, London, WC1E 7JE, United Kingdom

## Abstract

The development of fluid transport systems was a key event in the evolution of animals and plants. While within vertebrates branched geometries predominate, the choriocapillaris, which is the microvascular bed that is responsible for the maintenance of the outer retina, has evolved a planar topology. Here we examine the flow and mass transfer properties associated with this unusual geometry. We show that as a result of the form of the choriocapillaris, the blood flow is decomposed into a tessellation of functional vascular segments of various shapes delineated by separation surfaces across which there is no flow, and in the vicinity of which the transport of passive substances is diffusion-limited. The shape of each functional segment is determined by the distribution of arterioles and venules and their respective relative flow rates. We also show that, remarkably, the mass exchange with the outer retina is a function of the shape of each functional segment. In addition to introducing a novel framework in which the structure and function of the metabolite delivery system to the outer retina may be investigated in health and disease, the present work provides a general characterisation of the flow and transfers in multipole Hele-Shaw configurations.

First described in man by Hovius in 1702^1^, the choriocapillaris serves multiple functions that include sustaining the photoreceptors, cells that have one of the highest metabolic rates of any cell of the human body^2^, filtering waste produced in the outer retina and regulating the temperature of the back of the eye^3^,^4^. Of the two types of transfer-region geometries found in plants and across animal phyla, the most common one consists of branched networks of vessels linking broadly distributed transfer regions. Examples in man include the retinal vascular tree and the cerebral vasculature. Unlike most vasculatures, the choriocapillaris has evolved a planar geometry, whereby transfers between capillaries and tissue occur across a planar interface^5^. It comprises a 10–30 *μ*m thick continuous and dense mesh of characteristically flat, wide and non-overlapping capillaries. The capillaries lie in a single plane and are supported by a rigid framework of intercapillary connective tissue^6^,^7^,^8^,^9^,^10^,^11^,^12^,^13^,^14^,^15^,^16^ (Fig. 1). Because of the optical filtering effect of the retinal pigment epithelium lying internally to this vascular plexus, the choriocapillaris is difficult to image *in vivo*. Indirect measurements and fluorescent dye angiography have shown that the choriocapillaris blood flow is one of the highest of any vascular bed of the human body and that it largely lacks autoregulation^3^,^17^. Flow through the choriocapillaris is, per unit mass, three to four times higher than that in the kidney^18^.

The unusual nature of the choriocapillaris anatomy and the difficulty of imaging it systematically in man has historically hindered the characterisation of its functional features. One of the consequences of this is that the importance of anatomical and functional changes in the choriocapillaris in diseases of the back of the eye has often been misunderstood or overlooked. These include age-related macular degeneration (AMD) and diabetic retinopathy (DR), two of the most prevalent causes of vision loss^19^,^20^. In AMD, both structural^21^,^22^,^23^ and functional^24^,^25^,^26^ changes have been reported in the choroidal vasculature. In DR, only structural changes have to date been examined^27^,^28^,^29^,^30^. A framework in which the linkage between structural and functional changes may be investigated is currently lacking. Such a framework requires a thorough understanding of the dynamics of the choriocapillaris. In this study we have sought to describe the fundamental properties of this most unusual and significant vascular bed.

## Results

### Characterisation of the choriocapillaris geometry

**Figure 1:**
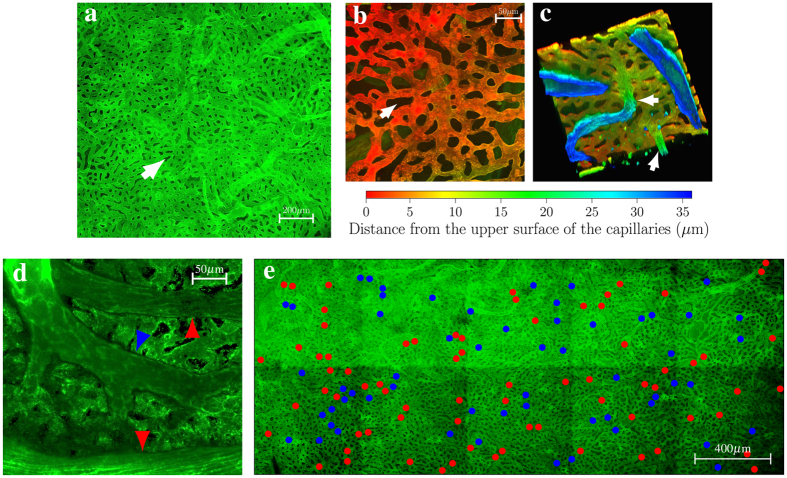
Vascular geometry of the choriocapillaris in man. Portions of human choriocapillaris in the posterior pole are shown from the retinal side in (**a**,**b**,**e**) and from the scleral side in (**c**,**d**). Shown in (**b**,**c**,**d**) are three-dimensional reconstructions of portions of choriocapillaris obtained by serially imaging the vascular plexus at different depths. The capillaries are separated by collagenous pillars spanning the thickness of the choriocapillaris (arrow, **a**, **b**). In (**c**), two venules are seen inserting into the plane of the capillaries at approximately a right angle (arrows). (**d**) Arterioles and venules inserting into the plane of the choriocapillaris may be differentiated by the morphology of their endothelial cells, which is characteristically more elongated in arterioles (red arrows) and more rhombic in venules (blue arrow). Shown in (**e**) is the distribution of arteriolar (red dots) and venular (blue dots) openings, which correspond to insertions of respectively feeding arterioles and draining venules into the outer surface of the choriocapillaris, over an extended portion of tissue taken from the posterior pole. The number of arteriolar openings is here *N*_*a*_ = 73; the number of venular openings is *N*_*v*_ = 59.

We first sought to characterise the vascular geometry of the choriocapillaris in man. To do so, portions of human choroid dissected from six human eyes were examined. The choriocapillaris essentially consists of two thinly-spaced planar parallel sheets serviced by arterioles and venules connected approximately perpendicularly to the outer sheet (Fig. 1c). The intercapillary connective tissue consists of a series of obstacles of various sizes and shapes spanning the width of the gap between the sheets (Fig. 1b). New approaches were developed to identify the locations of arteriolar blood supply and venular drainage over the capillary bed. These correspond to points of insertion of outer choroidal vessels into the outer surface of the plexus, and were identified by serially imaging extended portions of tissue (from tissue samples of typical dimensions 1 cm × 2.5 cm) transversely over its thickness, which varied between 10 and 20 *μ*m^31^, using confocal microscopy. In the outermost plane of the choriocapillaris, the connections between feeding and draining vessels and the plexus appeared as round openings typically 10–25 *μ*m in diameter^15^. Each opening was individually classified as arteriolar or venular from the nature of the intermediate choroidal vessel servicing it. Arterioles and venules were distinguished according to several criteria. The first was endothelial cell morphology, which was characteristically more elongated in arterioles and comparatively more rhombic in venules (Fig. 1d). Second, since the veins draining the choroidal vasculature – the vortex veins – and the posterior ciliary arteries supplying it insert in the eye at distinct locations, arterioles and venules typically travel and branch in different directions and at different angles. They could therefore be readily followed as they branched from larger vessels and inserted into the choriocapillaris. This made it possible to map arteriolar and venular openings (Fig. 1e). Both arteriolar and venular openings were found to appear in clusters of two or more. This clustering often resulted from the branching of arterioles and venules into two or more short terminal segments a few micrometers from the plane of the choriocapillaris; however, clusters sometimes also comprised branches arising from distinct arterioles or venules.

### Segmentation of the blood flow

**Figure 2:**
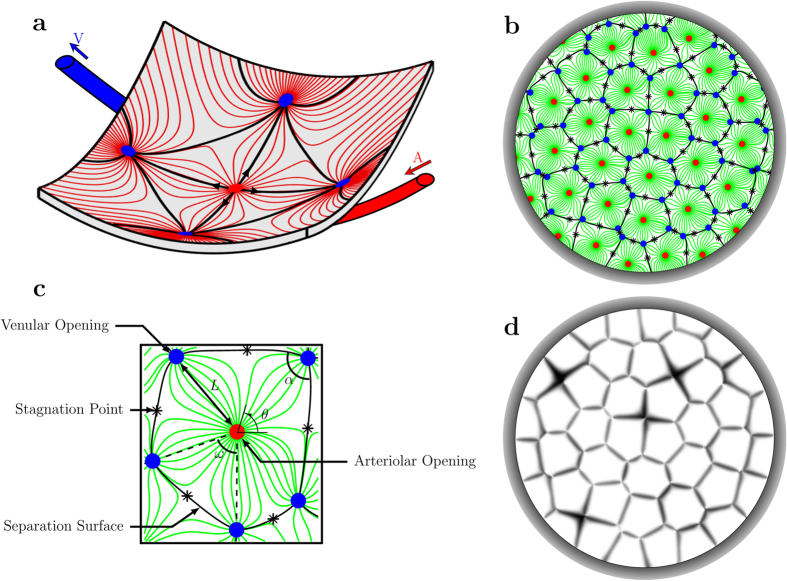
Geometrical segmentation of the choriocapillaris blood flow and its visualisation. A schematic of the streamline pattern in the mid-plane of the choriocapillaris is shown in (**a**). Arteriolar and venular openings are represented as respectively red and blue dots. Streamlines of the blood flow in the mid-plane of the choriocapillaris set by a random distribution of arteriolar and venular openings are plotted in (**b**). The separation surfaces of the flow field, which delineate functional vascular segments, are plotted as a series of connected black lines. Stagnations points are represented as asterisks. An individual functional vascular segment taken from (**b**) is represented in (**c**) and parameters of the model are indicated. The number of sides of the functional vascular segment is here 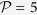. The vicinity of the separation surfaces takes a comparatively prolonged time to be reached by a passive dye filling the flow domain, as shown in (**d**) (Video S2). This property may be harnessed to visualise the segmentation of the blood flow.

The blood flow in the choriocapillaris is contained in the spherical curved plane of the choroid (Fig. 2a). Arteriolar and venular openings correspond to sources and sinks, respectively, in the flow field. Collagenous posts that separate capillaries may be modelled as non-overlapping circular extrusions of the flow domain in the direction perpendicular to the plane of the choriocapillaris. In the thin choriocapillaris, the blood flow is dominated by viscous effects and can be described everywhere other than within a short distance from the arteriolar and venular openings by a continuum model based on lubrication analysis. Streamlines describe the direction in which blood travels in the choriocapillaris. Robust geometrical theorems can be applied to analyse the topological features of the streamlines that lie in the mid-plane of the choriocapillaris; this technique has been applied to characterise the topology of inertially dominated flows over the surfaces of aeroplane wings and mountains^32^,^33^ but not low Reynolds number flows. It follows from the work of Leonhard Euler in 1758^34^ that the presence of arteriolar and venular openings over the spherical surface of the choriocapillaris generates nodal points in the streamline pattern of the flow, which leads to the appearance of saddle – or stagnation – points. At the stagnation points, the local velocity of blood is zero. The number of nodes, comprised of the number of arteriolar openings *N*_*a*_ (see Table 1 for a list of parameters) and venular openings *N*_*v*_, and the number of stagnation points *N*_*s*_ are related through (see Methods for proof)


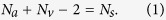


Further characterisation of the essential geometrical quality of the flow field can be achieved by using Poincare’s 1881 theory of continuous dynamical systems^35^ (and Bendixson’s early twentieth century extension^36^). The flow field set by a distribution of arteriolar and venular openings is decomposed into a finite number of subsets – here called functional vascular segments – delineated by separation surfaces joining two venular openings (Fig. 2b). The stagnation points of the flow field lie on the edges of these subsets. Importantly, there is no flow of fluid across these edges. The separation surfaces are not physical boundaries but arise as a result of the blood flow pattern in the choriocapillaris. They form the interface between the blood flowing from neighbouring arteriolar openings.

### Visualisation of the segmentation of the blood flow

**Figure 3:**
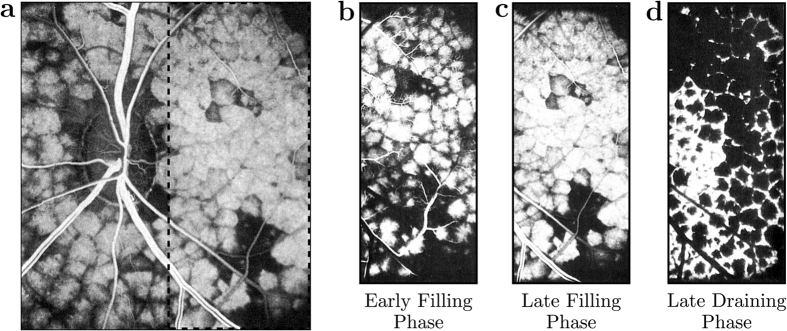
Fluorescent dye angiography of the choriocapillaris, taken with permission from Hayreh^62^,^63^. The scale bars are missing from the original publication. The mosaic pattern observed during the filling of the choriocapillaris with a passive fluorescent dye is shown in (**a**). Images (**b**–**d**) show the segmentation pattern at different time points as the passive dye fills (**b**,**c**) and drains (**d**) the choriocapillaris within the area delineated in (**a**). The fluorescent units forming the mosaic displayed in (**a**) have been described as ‘functional lobules’, and consist of one or a group of functional vascular segments as defined in the present work.

The velocity in the direction perpendicular to a separation surface tends to zero with distance to the surface^37^ while the travel time of a fluid particle on a separation surface is infinite so that the passive transport in their vicinity is diffusion-limited. This property may be harnessed to visualise the segmentation of the blood flow by filling the choriocapillaris through the arteriolar openings with a passive dye (Fig. 2c). The central part of each vascular segment rapidly fills but the edges next to the stagnation surfaces take comparatively longer to fill (Video S2). This behaviour has been observed whenever the transport of a dye has been visualised in the choriocapillaris (such as during dye angiography) *in vivo*, with various imaging techniques^17^,^38^,^39^,^40^ (Fig. 3). An explanation of the presence of these vascular segments has eluded investigators ever since it was first observed, with many believing that the filling pattern resulted from the presence of physical barriers between independent entities called functional lobules^15^,^17^,^38^,^39^,^40^. In fact, functional lobules correspond to one or a group of functional vascular segments as defined here.

### Characteristics determining the shape of functional vascular segments

A geometrical description of the linkage between anatomical and functional characteristics of the choriocapillaris was sought by examining the relation between the number of arteriolar and venular openings, their ratio (which changes with location in the eye), their respective flow rates and the shape of functional vascular segments. The ratio between arteriolar and venular openings servicing a subset of the flow field may be determined by measuring the interior angles at the vertices of a functional vascular segment (see Fig. 2c and Methods). In cases where the separation surfaces delineating a vascular segment may be approximated by planes, the segment has the aspect of a prism, and the ratio of arteriolar to venular openings satisfies (see Methods for the proof)


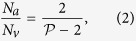


where 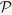 is the number of sides of the functional segment. The flow at an arteriolar opening may be decomposed into a finite number of portions drained by distinct venular openings. The contribution of each of these venular openings is described by the interior angles at the vertices of functional vascular segments. By harnessing this property, and by denoting *Q*_*a*_ the volumetric flow rate at an arteriolar opening and *Q*_*v*_ the volumetric flow rate exiting the functional vascular segment through the venular openings, the average arteriolar flow rate (here denoted 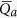) satisfies (see Methods for the proof)


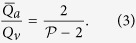


This relation does not require any assumption on the flow rates of arteriolar and venular openings. If the flow rate at each venular opening draining vascular segments is comparable, then 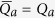, and (3) may be rewritten


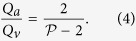


The same relation is obtained if the interior angles at the vertices of the functional vascular segment are equal. If the separatrices may be approximated by straight lines, then (2) and (3) yield


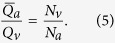


Relations (2) and (3) demonstrate that the shape of functional vascular segments as described by the parameter 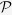 is determined by the relative distribution of arteriolar and venular openings and their relative flow rates. Interestingly, while the location of venular openings determines the position of the vertices of functional vascular segments, (3) and (4) show that the position of arteriolar openings within the interior of each segment may not be inferred from geometrical considerations alone. The presence of non-axisymmetric flows at arteriolar openings and variations in the relative contribution of venular openings to the drainage of vascular segments must be considered to estimate the position of arteriolar openings from the shape of functional vascular segments.

Importantly, (2) and (3) show that the occlusion of arteriolar or venular openings or a reduction in their relative flow rates changes the shape of functional vascular segments. More generally, by linking the interior angle at the vertices of functional vascular segments to arteriolar and venular flow rates, the present analysis provides tools to assess local changes in arteriolar and venular flow rates. It also shows how the difficulty of observing and quantifying aspects of the choriocapillaris in man *in vivo* can, to some extent, be circumvented by making use of the geometrical quality of the blood velocity field. The ratio between arteriolar and venular openings and their relative volumetric flow rates may for instance be estimated by simply counting the number of sides of functional vascular segments as observed during dye angiography.

### Relation between the shape of functional vascular segments and the mass exchange with the outer retina

**Figure 4:**
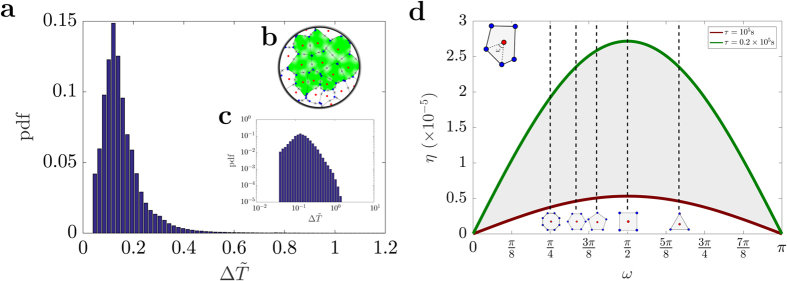
Relation between the shape of functional vascular segments, the corpuscle travel time and mass extraction. Shown in (**a**,**c**) is the probability density function (pdf) of the travel time of a corpuscle (here denoted 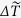) calculated for ten adjacent functional vascular segments of a randomly generated distribution of arteriolar and venular openings (plotted in (**b)**). In (**d**), the evolution of the mass extraction *η* is plotted as a function of the angle *ω* between an arteriolar opening and two consecutive venular openings feeding and draining the same functional vascular segment (see Methods). Regular polygons associated with certain angles are indicated. The shaded area corresponds to 0.2 × 10^5^ s ≤ *τ* ≤ 10^5^ s, which concurs with an extraction rate of between 1 and 5% per volume of blood^42^,^43^,^44^.

The most important function of the choriocapillaris is to sustain adequate bidirectional mass exchange to deliver the metabolites sustaining the photoreceptors and to remove waste from the outer retina. As a result of the configuration of the choriocapillaris, this function is fulfilled within a confined space without causing any scattering of the light travelling towards the photoreceptors. In order to assess this function in relation to the geometrical segmentation of the blood flow, the mass exchange between retina and choriocapillaris was examined. The mass extraction is defined as the difference in the average concentration of a compound in blood entering and leaving the choriocapillaris. It is a function of the travel time of corpuscles responsible for carrying elements transferred to the outer retina from the choriocapillaris, which is a measure of the time that they spend in the plane of the capillary bed after being released at an arteriolar opening and before being removed at a venular one (Fig. 4a–c). Therefore, mass extraction was investigated using a Lagrangian transport model of corpuscles. Within a functional vascular segment, the mass extraction (here denoted *η*) is related to the average corpuscle travel time through the relation


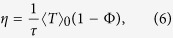


where Φ is the volume fraction of tissue occupied by the interconnective tissue (0.25 ≤ Φ ≤ 0.5^41^), 〈*T*〉_0_ is the average travel time of a corpuscle between an arteriolar and a venular opening in the absence of interconnective tissue (〈*T*〉_0_ × (1 − Φ) ~ 0.12 s^39^), and 1/*τ* is a mass transfer coefficient. The value of *τ* is a function of the velocity of blood, the diffusivity of the compound considered and the gradient of concentration between blood and the surrounding tissue. In the choriocapillaris, the oxygen extraction rate is between less than one percent and five percent per volume of blood^42^,^43^,^44^. The glucose extraction rate lies within a similar range^42^. It may therefore be estimated that for these two compounds, *τ* ≈ 0.2–1 × 10^5^ s (see Methods). The mass extraction from a functional vascular segment also satisfies (see Methods).


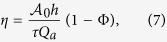


where 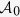 is the surface area of the functional vascular segment in the absence of intercapillary collagenous posts, which is set by the relative distribution of arteriolar and venular openings and their relative flow rates; *Q*_*a*_ is the volumetric flow rate at the arteriolar opening supplying it and *h* is the thickness of the choriocapillaris. Relation (7) demonstrates the interdependence between anatomical characteristics of the choriocapillaris (through *h* and Φ) and functional ones (through *Q*_*a*_, 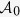, *τ*) in determining mass exchange between blood and tissue.

This analysis shows that the experimentally observed variability in the shape of functional vascular segments reported during angiography of the choriocapillaris^15^,^17^,^38^,^39^,^40^ results from variations in the distribution of arteriolar and venular openings and their respective flow rates. However, the dependence between the shape of functional vascular segments and mass extraction has yet to be established. This dependence may be characterised by examining the relation between the corpuscle travel time, mass extraction and the shape of functional vascular segments in the limit, where separation surfaces may be approximated by planes (Fig. 4d and Methods). As shown in Fig. 4d, mass exchange is a function of the shape of functional vascular segments, and has a maximum for regular square-shaped segments.

## Discussion

As a result of the planar geometry of the choriocapillaris and the way in which it receives and drains blood, the blood flow through the vascular plexus is decomposed into a tessellation of experimentally observable^17^,^38^,^39^,^40^ functional vascular segments delineated by separation surfaces across which there is no flow. The passive transport in the vicinity of the separation surfaces of the flow field is diffusion-limited so that the segmentation of the blood flow may be observed by filling and draining the choriocapillaris with a passive dye. The shape of functional vascular segments is set by the distribution of arteriolar and venular openings and their respective relative flow rates, and is a determinant of the mass exchange with the outer retina.

The aim of the present work has been to describe the fundamental properties of the blood flow and transport associated with the geometry of the choriocapillaris, and to assess them in relation to the function that the capillary plexus fulfils in the back of the eye. As such, some of the complexity associated with the flow of blood in capillaries and the mass exchange between blood vessels and tissue has been excluded from the study. The Lagrangian model assumes that corpuscles travel at the mean blood velocity. While the dimensions of erythrocytes and the diameter of choriocapillaris vessels make this assumption legitimate^45^, the velocity of corpuscles of dimensions different from erythrocytes may be slightly different than the mean bulk velocity. In this case, the travel time defined in the present analysis represents only a metric of the time that they spend in the plane of the choriocapillaris. The analysis has been carried out at the scale of clusters of functional vascular segments, which was necessary to prove the functional segmentation of the blood flow and transport of a passive dye. Although the averaged effect of the intercapillary septal posts on the blood flow and mass exchange have been included in the study, an analysis at a smaller scale is necessary to describe their impact on the trajectory of corpuscles^46^ and investigate the characteristics of the mass exchange in their vicinity. While the segmentation of the blood flow described here is qualitatively consistent with dye angiography of the choriocapillaris, a quantitative validation of (6) and (7) is difficult to obtain. Typical values for the coefficient *τ* have been estimated using oxygen and glucose extraction rates, which are the only substances for which data were available. Nevertheless, (6) and (7) may be used to compare the relative blood flow and mass transfer of functional vascular segments, and therefore represent valuable tools to detect and monitor microvascular changes in the choriocapillaris. It is important to stress that the rate of exchange between blood and tissue is substance-specific. Exchange between the choriocapillaris and the retina are also crucially a function of the types of transport process involved, which may have either or both active and passive components. While the conclusions of this paper have fundamental implications for the transport and delivery of compounds delivered to the retina at the mesoscale, it should be complemented with substance-specific analyses to describe exchange at a smaller scale.

Despite these limitations, the present analysis has important implications for the understanding of the structure and physiology of the choriocapillaris. The segmental nature of the choriocapillaris blood flow observed during dye angiography^17^,^38^,^39^,^40^ has historically been considered incompatible with the continuity of the vascular plexus. However, physical boundaries between independent anatomical vascular units could not be found, which led to ongoing controversy and misconceptions regarding the structure and function of the choriocapillaris^15^,^47^. The present work demonstrates that physical boundaries are in fact not necessary to observe a segmentation of the blood flow and passive transport, and that the continuity of the vascular plexus is perfectly compatible with angiographic observations.

Interestingly, the present analysis suggests that the distribution of arteriolar and venular openings and their respective flow rates – and therefore the shape of functional vascular segments – may be tuned to locally adjust mass exchange with the outer retina. The ratio between arterioles supplying the vascular plexus and the venules draining it decreases significantly from the submacular area to the periphery of the eye^14^,^15^,^48^. The impact of this decrease on the shape of functional vascular segments may be estimated by approximating the separatrices of the flow field by straight lines and by using (2). As *N*_*a*_/*N*_*v*_ is reduced, the number of sides of functional vascular segments must increase. At constant flow rate this would suggest that the mass extraction is comparatively lower in the periphery compared to the submacular area (Fig. 4d). This is consistent with the cellular organisation of the retina over the eye as the density of rod and cone photoreceptors is maximal in the submacular area and decreases towards the periphery^49^. From (2) and (5), variations in the shape of functional vascular segments may also alleviate the consequences of discrepancies in arteriolar flow rates over the eye. In effect, the present work demonstrates the existence a necessary level of regularity between the metabolic requirements of the outer retina, the relative distribution of arteriolar and venular openings and their respective relative flow rates. It is at present unclear if this regularity arises from feedback mechanisms between choroid and retina occurring during the embryonic development of the choriocapillaris. Our analysis also shows that there is an optimum between the shape of functional vascular segments and the mass exchange rate between choriocapillaris and outer retina (Fig. 4d).

Methods to assess the function of the choriocapillaris in man are currently limited. The present work introduces novel methods to identify arteriolar and venular openings in the human choriocapillaris and to explore the linkage between their distribution and the metabolite supply to the outer retina. It also shows how variations in the shape of functional vascular segments may be harnessed to assess and monitor the mass delivery to the outer retina *in vivo* on a per-patient basis. Past animal studies have shown that the travel time of passive dyes and the surface area of one or a group of functional vascular segments may be measured by fluorescent dye angiography^39^. From our analysis, these parameters would allow for an assessment of the efficiency of the metabolite delivery to portions of the outer retina. This work will hopefully pave the way to the development of more effective imaging and diagnostic techniques for pathologies of the back of the eye.

The mathematical approach developed in this paper finds application in flow systems with geometries similar to the choriocapillaris. Despite being found in various invertebrates and vertebrates^5^, vascular geometries involving planar mass transfers have seen little to no quantitative analysis. From the aspect of *in silico* systems, the present work represents a general characterisation of the flow and mass transport in planar multipole Hele-Shaw configurations. Planar source flows^50^,^51^,^52^,^53^ or flows set by multipoles^54^,^55^ in Hele-Shaw configurations have mostly been investigated with free boundaries. The passive transport in this type of configuration has seldom been examined. Quadrupoles composed of two inlets and two outlets have been used experimentally to generate a floating gradient of concentration in the vicinity of a stagnation point at very high flow rates^55^. More research is needed to characterise many aspects of Hele-Shaw flows. For instance, the stretching of the trajectories of corpuscles as they travel from an arteriolar to a venular opening suggest important mixing in the plane of the choriocapillaris and is likely to enhance the reactivity of solutes contained in blood^56^. On the basis of our findings, planar microfluidic multipoles may also be designed to adjust exchange rates locally or to alleviate spatial variations in inlet pressures.

## Methods

### Whole-choroid immunohistochemistry

The protocol presently described adhered to the tenets set forth in the Declaration for Helsinki regarding research involving human tissue. Six human donor eyes with no known ocular disease were obtained from the Moorfields Eye Hospital Eye Bank, London, under Full Local Research Ethics Committee approval and appropriate informed consent. Maps of arteriolar and venular openings were obtained for samples extracted from four of these eyes. The eyes were fixed in 4% paraformaldehyde 24 to 50 hours after death and kept in saline in a fridge at 4 °C for up to four years. Samples were dissected and washed with 0.1 M phosphate buffer saline (PBS). The retinal pigment epithelium (RPE) was detached from Bruch’s membrane by incubating the tissue with 0.5% Trypsin + EDTA (Life Technologies Ltd, UK) at room temperature and gently brushing the RPE under a dissection microscope. The tissue was incubated for one hour with fluorescein isothiocyaniate labelled UEA-I (Vector Laboratories, USA), washed in PBS and then in tris buffered saline. The whole-mounts were analysed by confocal microscopy using a Zeiss 710 LSM microscope (Carl Zeiss, Germany). The imaging of large portions of tissue required the sequential imaging of adjacent tiles and their subsequent stitching in post-processing, which was carried out using the Zen software (Carl Zeiss, Germany). The software was also used to perform three-dimensional reconstructions of portions of the vasculature.

### Mathematical model for the blood flow

Blood was modelled as a Newtonian fluid with constant viscosity. The Reynolds number (*Re*) in the choriocapillaris, calculated with the blood velocity measured *in vivo*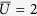 mm.s^−1^ 17,57,58, is of the order of 0.1, so that the blood flow is viscously dominated and satisfies *Re*(*h*/*L*)^2^ ~ 10^−5^, where *L* is a characteristic distance between an arteriolar and a venular opening and *h* is the thickness of the choriocapillaris. By defining the cross-sectionally averaged velocity in the plane of the choriocapillaris as


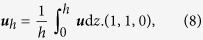


where *u* represents the velocity in the thin layer of the capillaries, and by denoting


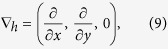


the velocity field is described everywhere but within a short distance from the arteriolar and venular openings by the lubrication equation


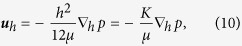


where *p* is the pressure, *μ* is the viscosity of blood and *K* = *h*^2^/12 is the permeability characterising the flow between parallel plates.

### Topological analysis of the flow field

The topological analysis of the flow field was carried out on a sphere. Collagenous pillars were modelled as non-overlapping discs of various diameters extruded from the sphere so that the surface considered consisted of a sphere with holes. By denoting *n*_*d*_ the number of holes, the Euler characteristic (here denoted *χ*) of the sphere with holes is expressed as^59^


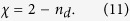


In topological terms, the arteriolar and venular openings correspond to nodes of the flow field and the streamlines of the flow correspond to orbits of the system. Each hole introduces two half-crosspoints. The indexes of the nodes (denoted *σ*_*n*_) and crosspoints (denoted *σ*_*s*_) therefore satisfy^59^


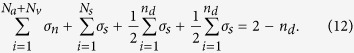


Since *σ*_*n*_ = 1 and *σ*_*s*_ = −1, this equation yields


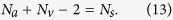


Relation (13) shows that the topological characteristics of the flow field in the presence and absence of holes are the same. Without loss of generality, Φ was taken to be zero, and the flow was investigated on the Riemann sphere identified with the extended complex plane 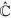. The flow at arteriolar and venular openings was modelled as respectively point source and sink flows. The complex velocity field satisfied


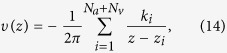


where *k*_*i*_ is the strength per unit length of the *i*th arteriolar (*k*_*i*_ > 0) or venular (*k*_*i*_ < 0) opening. Since the system (14) is described by a rational vector field, the Riemann sphere is decomposed into a finite number of open sets, which are each invariant under the system (14). Each subset is composed of the set of periodic trajectories connecting two nodes of the flow field, an arteriolar and a venular opening. The complement of these open sets consists of a finite number of trajectories, for which the flow is not analytic in at least one direction^37^. They correspond to asymptotes of the trajectories of each subset, connect a crosspoint point to two nodes of the flow field and satisfy the definition of a separatrix^60^. At the stagnation points, the open subsets form hyperbolic sectors. The set of separatrices enclosing an inlet formally delineate a subset of the flow domain, where all the possible trajectories of a fluid particle released at the inlet are contained.

### Simulation of the filling and flushing of the choriocapillaris with a fluorescent dye

The fluorescence of a dye in whole blood varies below a maximal concentration quasi-linearly with its concentration; therefore, fluorescent dye angiography of the choriocapillaris was simulated by visualising the evolution of the concentration of a passive scalar filling and being flushed from the flow domain. The transport of a passive scalar characterised by a mass concentration *C* and a constant diffusivity *D* independent of *C* is described by the advection-diffusion equation, i.e.


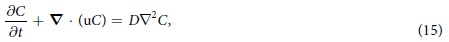


where ***u*** is the velocity field. Here, the advection-diffusion equation is considered in two dimensions, with


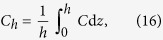


so that the advection-diffusion equation becomes


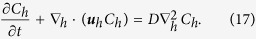


The velocity field *u*_*h*_ was determined analytically using (14). In order to simulate the filling and flushing of the choriocapillaris with a fluorescent dye, a double time-dependent Heaviside was imposed simultaneously at all the arteriolar openings, i.e.


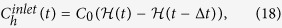


where *C*_0_ is the concentration imposed at each inlet, 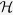 is the Heaviside function and *t* is the time variable. Over the rest of the domain, *C*_*h*_(*t* = 0) = 0 was imposed. The domain was bounded by imposing a no-flux boundary condition over a circle enclosing the flow domain. The advection-diffusion equation was solved numerically using the finite element method.

### Relation between the ratio of arteriolar to venular openings and the shape of vascular segments

Any arteriolar opening feeds one functional vascular segment only; however, venular openings empty adjacent segments. Therefore, only a portion of them participates in the draining of each functional vascular segment. This contribution is described by the interior angle at the vertices of each vascular segment *α*_*k*_. The number of venular openings satisfies


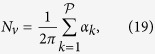


where 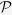 is the number of edges delineating each functional vascular segment; therefore,


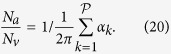


In the limit, where the separation surfaces are approximated by planes, functional vascular segments form polygonal prisms. The number of nodes *σ*_*n*_ present within any polygonal segment is


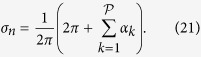


Between each venular opening is a crosspoint of the flow field. Within the domain delineated by the boundaries of the functional vascular segment, the number of nodes and crosspoints *σ*_*s*_ satisfy^32^


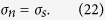


There is one crosspoint for each side of the polygon. Since for each of these points the angle subtended by the crosspoint on the side of the vascular segment is *π*, *σ*_*s*_ satisfies


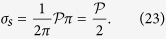


Combining equations (21) and (22) yields


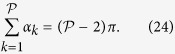


The ratio between arteriolar and venular openings is therefore


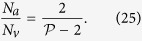


### Relation between the ratio of arteriolar to venular flow rates and the shape of a vascular segments

As a result of the segmentation of the flow field all the possible trajectories of a fluid element entering the choriocapillaris at an arteriolar opening are contained in a functional vascular segment drained by a finite number of venular openings. The flow at an arteriolar opening is decomposed into a finite number of portions drained by distinct venular openings. The flow rate at an arteriolar opening (here denoted *Q*_*a*_) may therefore be expressed as


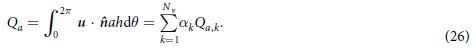


where *Q*_*a*,*k*_ describes the portion of blood drained at the *k*th venular opening and *a* is the radius of the opening. At any arteriolar opening, the average flow rate may be expressed as


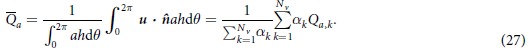


By denoting *Q*_*v*_ the volumetric flow rate exiting a segment, imposing mass conservation yields


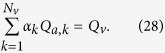


Since


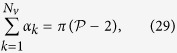


(27) may be rewritten


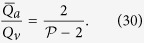


This expression is general and does not require any assumption on the flow rates at arteriolar or venular openings. If each venular opening has the same contribution to the drainage of the functional vascular segment, then from (27), 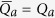, and


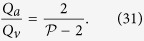


If all the angles *α*_*k*_ are equal the same relation is obtained. In the limit, where the separatrices delineating the functional vascular segment may be approximated by a straight line, (30) and (25) yield


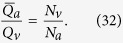


### Mass extraction model

Elements transported in the choriocapillaris and exchanged with the outer retina are either corpuscles, are carried by corpuscles or are dissolved in plasma. The mass transfer from the choriocapillaris to the surrounding tissue is largely determined by the time that these elements spend in the plane of the capillaries. In capillaries, the velocity of corpuscles is a function of their size and the diameter of the vessels that they travel in; it is not always equal to the mean bulk velocity ***u***_*h*_. However, given the dimensions of erythrocytes and the diameter of choriocapillaris vessels, it can in the present analysis be assumed that their velocity is equal to ***u***_*h*_^45^. Their transport is therefore described by their travel time between an arteriolar and a venular opening. Within a functional vascular segment, by making the velocity field ***u***_*h*_ dimensionless with respect to 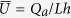 and scaling all distances to the average distance between the arteriolar and venular openings, the dimensionless travel time of a corpuscle traveling at the mean bulk velocity ***u***_*h*_ is defined as


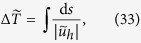


where the integral is taken over a streamline and 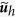 is the dimensionless velocity. The velocity of corpuscles of dimensions different from erythrocytes may be larger or smaller than the mean bulk velocity ***u***_*h*_, in which case (33) represents only a metric of the time that they spend in the plane of the choriocapillaris. Mass extraction from the choriocapillaris is largely driven by gradients of concentration in the direction perpendicular to the plane of the choriocapillaris. Therefore, the transfer of an element from the choriocapillaris to the surrounding tissue is described by a first order exchange process proportional to the difference in concentration between blood *C*_*h*_ and the surrounding tissue *C*_*t*_. By taking *C*_*t*_ = 0 without loss of generality, and assuming that the concentration field in the choriocapillaris is stable over time, the concentration along the path taken by a corpuscle satisfies:


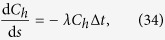


where *λ* is the rate of exchange between the corpuscle and the surrounding tissue. The concentration is made dimensionless relative to the concentration at the arteriolar opening 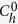, which is assumed to be axisymmetric. Lengths and time are scaled relative respectively to the characteristic distance *L* and time scale 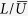. The dimensionless equation for mass extraction becomes:


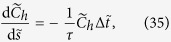


where 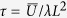 represents the ratio between the momentum of blood and the surface over which extraction occurs. The concentration at the venular opening where blood is drained is:


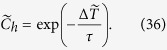


The average concentration in blood leaving a vascular segment is obtained by integrating (36) over all the corpuscles released at an arteriolar opening. By denoting *θ* the angle at the arteriolar opening, the average concentration satisfies


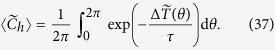


The average mass extraction from a functional vascular segment (denoted *η*) is


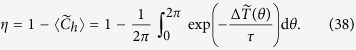


It is shown in Supplementary Information 1 that (38) is finite. The range of *τ* may be estimated by considering the typical oxygen and glucose extraction from the choriocapillaris and the average travel time measured during angiography. Since, using equation (38):


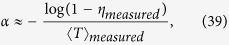


where *η*_*measured*_ and 〈*T*〉_*measured*_ are respectively the extraction rate and average residence time measured *in vivo*, *τ* satisfies:


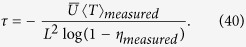


Typically, 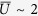
mm.s^−1^ 17,39,57,58 and *L* ~ 600 *μ*m^40^,^61^. The oxygen extraction rate was reported to be between 1 and 5% per unit of volume of blood^42^,^43^,^44^. The glucose extraction rate lies within a similar range^42^. Since the average travel time in a functional lobule was found to be about 118 ms, it may be inferred that


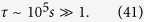


A Taylor expansion to the second order of (37) for 1/*τ* ≪ 1 yields:


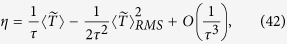


where


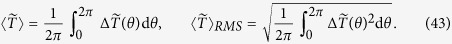


The first term corresponds to the average travel time while the second term is a measure of the mechanical dispersion of the travel time. Given the value of *τ*, the mechanical dispersion term is negligible, and (42) may be written (see Supplementary Information 1 for a comparison between exact and asymptotic expressions)


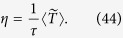


Since^46^


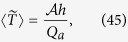


(44) yields


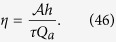


The effect of the collagenous posts may be made explicit in (44) and (46) by introducing the volume fraction of tissue occupied by the intercapillary connective tissue, which is here denoted Φ. Since the area of a functional vascular segment may be written 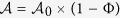, where 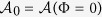, (44) and (46) may be written


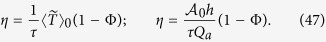


### Relation between the shape of functional vascular segments and mass extraction

The relation between the shape of functional vascular segments and mass extraction was examined by considering single vascular segments and varying their shapes. The flow rate at the arteriolar opening supplying the segment was kept constant. The pressure at each of the venular openings draining it was taken to be constant and for simplicity equal to zero. Under these assumptions, the functional vascular segment has the aspect of a prism, and may be decomposed into juxtaposing triangular portions characterised by an apex angle *ω* (Fig. 4d). For simplicity, it was assumed that the triangular portions were isosceles^46^. Under these assumptions, *ω* is the parameter describing the shape of the functional vascular segment.

If the functional vascular segment is taken to be a regular polygon, then *ω* satisfies


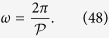


From this relation, it may for instance be inferred that *ω* = *π*/2 characterises a square. The regular polygons associated with remarkable values of *ω* are indicated in Fig. 4d.

## Additional Information

**How to cite this article**: Zouache, M. A. *et al*. Form, shape and function: segmented blood flow in the choriocapillaris. *Sci. Rep.* **6**, 35754; doi: 10.1038/srep35754 (2016).

## Supplementary Material

Supplementary Information

Supplementary Video S2

## Figures and Tables

**Table 1:** List of parameters describing the morphology and physiology of the choriocapillaris used in the model.

Parameter	Notation
Morphological parameters	
Number of arteriolar openings	*N*_*a*_
Number of venular openings	*N*_*v*_
Ratio of arteriolar to venular openings	*N*_*a*_/*N*_*v*_
Distance between arteriolar and venular openings	*L*
Thickness of the choriocapillaris	*h*
Volume fraction of tissue occupied by collagenous pillars	Φ
Physiological parameters	
Number of stagnation points	*N*_*s*_
Arteriolar flow rate	*Q*_*a*_
Venular flow rate	*Q*_*v*_
Number of sides of functional vascular segments	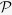
Surface area of functional vascular segments	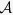
Travel time of a corpuscle	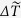
Average travel time of corpuscles within a functional vascular segment	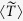
Mass exchange coefficient	1/*τ*
Mass exchange	*η*
