# Supplementary material for: Form, shape and function: segmented blood flow in the choriocapillaris
Source: Sci Rep. 2016 Oct 25;6:35754. doi: 10.1038/srep35754 (PMC5078844; doi:10.1038/srep35754)
Supplement: Supplementary Information [file srep35754-s1.pdf]

Form, shape and function: segmented blood flow in the choriocapillaris

M. A. Zouache[1,*], I. Eames[2], C. A. Klettner[2], and P. J. Luthert[1]

[1]University College London, Institute of Ophthalmology, London, EC1V 9EL, United Kingdom
[2]University College London, Department of Mechanical Engineering, London, WC1E 7JE, United Kingdom
[*]moussa.zouache.10@ucl.ac.uk

Supplementary Information 1: asymptotic expression of the mass extraction for large values of τ

From the Methods section, the mass extraction from a functional vascular segment is expressed as

$$\eta = 1 - \langle \tilde{C}_h \rangle = 1 - \frac{1}{2\pi}\int_0^{2\pi} \exp\left(-\frac{\Delta\tilde{T}(\theta)}{\tau}\right) \mathrm{d}\theta, \qquad (1)$$

where θ represents the angle at which the corpuscle is released at an arteriolar opening and τ is fixed. The aim of this supplementary information section is to show that the integral in the expression of η is finite, and that a Taylor expansion of (1) for large values of τ gives an accurate solution.

The typical evolution of the travel time of a corpuscle released at an arteriolar opening as a function of θ is shown in Fig. 1. On the streamline connecting the arteriolar opening to a stagnation point the travel time is infinite. Adjacent to the stagnation streamline, the travel time has a logarithmic dependence on θ. This singularity is integrable, so that the average travel time over a functional vascular segment is finite[1]. In the presence of intercapillary connective tissue, here modelled as a series of circular obstacles spanning the thickness of the choriocapillaris, the travel time has a local logarithmic divergence relative to the centre of the obstacle which is also integrable and finite[2]. It follows that the integral term in (1) is finite.

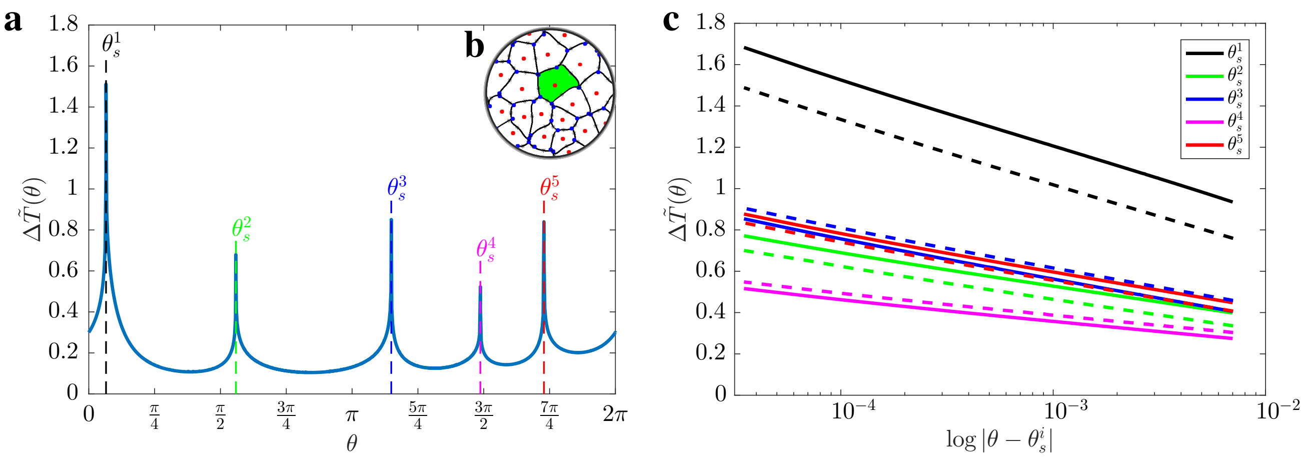

Figure 1. Evolution of the travel time of a corpuscle within a functional vascular segment as a function of the angle at which it is released from an arteriolar opening. Shown in (**a**) is the travel time distribution for corpuscles released at the arteriolar opening feeding the functional vascular segment highlighted in (**b**). The angle at which corpuscles are released is here denoted θ. The angles corresponding to the stagnation streamlines are denoted θ_s^i, where $1 \leq i \leq 5$. In (**b**), the distribution of arteriolar (red dots) and venular openings (blue dots) was randomly generated. The travel time distribution in the vicinity of the stagnation streamline is plotted on a semilogarithmic scale in (**c**). The plain and dashed lines correspond respectively to $(\theta - \theta_i^s) > 0$ and $(\theta - \theta_i^s) < 0$. The graph shows that the travel time of corpuscles travelling adjacent to the stagnation streamline has a logarithmic dependence on θ.

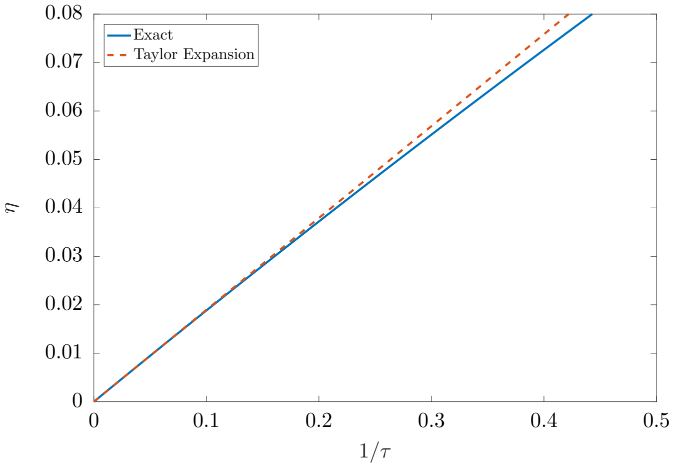

Figure 2. Evolution of the exact and asymptotic expressions of the mass extraction (η) as a function of $1/\tau$. The exact value of η and its asymptotic expression coincide for large values of τ.

It was shown in the Methods section that $\tau \sim 10^5 s \gg 1$ so that to the first order, η may be expressed as

$$\eta = \frac{1}{\tau}\langle\tilde{T}\rangle + O\left(\frac{1}{\tau^2}\right), \tag{2}$$

where

$$\langle\tilde{T}\rangle = \frac{1}{2\pi}\int_0^{2\pi}\Delta\tilde{T}(\theta)\mathrm{d}\theta. \tag{3}$$

A comparison of (1) and (2) is plotted in Fig. 2. The Taylor expansion gives accurate values for $\tau \geq 10$.

References

1. Zouache, M. A., Eames, I. & Luthert, P. J. Blood flow in the choriocapillaris. *J. Fluid Mech.* **774**, 37–66 (2015).

2. Eames, I., Belcher, S. E. & Hunt, J. C. R. Drift, partial drift and darwin's proposition. *J. Fluid Mech.* **275**, 201–223 (1994).

Supplementary Information 2: description of Video S2

Simulation of dye angiography of the choriocapillaris

Ocular fluorescent dye angiography consists of the visualisation and imaging of the transport of a fluorescent dye initially injected in the general vasculature through ocular blood vessels. Under illumination at a dye-specific wavelength, the dye fluoresces and emits light at a wavelength different from the one used for excitation; this emitted light may then be captured by an optical system placed close to the cornea. Below a maximum concentration, the fluorescence of the dye varies quasi-linearly with its concentration in blood; therefore, the relative fluorescence observed during an angiogram is determined by the concentration field of the dye.

Angiography of the choriocapillaris was simulated by filling flow domains simultaneously through the arteriolar openings with a dye (modelled as a passive scalar) until the domain was fully filled (uniform arteriolar concentration over the flow domain). The dye was then flushed through the venular openings by setting the concentration at the arteriolar openings to be zero. Video S2 was generated by extracting the concentration field at a series of time points and plotting its contour on a grayscale.

Characteristics of video S2

The flow domain in video S2 contains $N_a = 44$ arteriolar openings and $N_v = 96$ venular ones and follows the distribution plotted in Fig. 2B. The velocity field was analytically determined by using equation (14) and by imposing $k_{i,source} = 0.1426$ for $1 \leq i \leq N_a$ and $k_{i,sink} = -0.0653$ for $1 \leq i \leq N_v$ so that the Péclet number was equal to $Pe = 240$. At $\tilde{t} = 0$, a concentration $\tilde{C}_h = 1$ was imposed at the arteriolar openings (filling phase). At $\tilde{t} = 15$, $\tilde{C}_h = 0$ was imposed at the arteriolar openings (flushing phase). The simulation was stopped when the dye was fully drained, at $\tilde{t} = 30$.
